# Urinary Total Protein as the Predictor of Albuminuria in Diabetic Patients

**DOI:** 10.5812/ijem.4236

**Published:** 2012-06-30

**Authors:** Sima Hashemipour, Maliheh Charkhchian, Amir Javadi, Ahmad Afaghi, Ali Akbar Hajiaghamohamadi, Ali Bastani, Fateme Hajmanoochehri, Amir Ziaee

**Affiliations:** 1 Metabolic Disease Research Center, Qazvin University of Medical Sciences, Qazvin, IR Iran; 2 Qazvin University of Medical Sciences, Qazvin, IR Iran

**Keywords:** Urinary Albumin, Urinary Protein, Diabetic Nephropathy

## Abstract

**Background:**

In order to detect nephropathy, measurement of total (24 hrs) urinary albumin or albumin/creatinin ratio in random urine samples is being recommended. But methods of albumin measurement are not available in all laboratories and also cost about 6 times more than that of urinary total protein measurement.

**Objectives:**

This Study was performed to determine appropriate cut off point in 24 hours urine total protein to diagnose micro- and macroalbuminuria in patients with diabetes mellitus.

**Patients and Methods:**

In this study, 204 patients with diabetes mellitus type I and II were selected. In collected 24 hours urine from patients, protein and albumin were measured by using Pyrogallol and Immunoturbidimetry methods, respectively.

**Results:**

Normoalbuminuri (albumin < 30 mg/24 hrs urine), microalbuminuri (albumin = 30-300 mg/24 hrs urine), and macroalbuminuri (albumin > 300 mg/24 hrs urine) were detected in 130, 51, and 23 patients, respectively. In 24 hrs urine collections, amounts of protein and albumin were compared to calculate cut off point of exerted protein for nephropathy diagnosis. cut off point of 73 mg/day for urinary total protein had appropriate sensitivity (94.5 %, CI = 91.4 % -97.6 %) and specificity (77.9 %, CI = 72.8 % -82.9 %) for microalbuminuria, while cut off point of 514 mg/day (sensitivity 95.7 %; specificity 98.9 %) was detected for diagnosis macroalbuminuria. Urine protein exertion of 150 mg/day that is currently considered as a normal value in most laboratory kits had a sensitivity of 73.1 % by which 30 % of microalbuminuric cases remained undiagnosed.

**Conclusions:**

Urinary total protein cut-off points of 73 mg/day and 514 mg/day were diagnostic for micro- and macroalbuminuria, respectively.

## 1. Background

Diabetes mellitus is the most common cause of renal failure in the world (1). Diabetic nephropathy with proteinuria, including albuminurinuria, occurs in about 20 – 40 % of diabetics from which a considerable cases may proceed to severe renal failure (2, 3)and also to cardiovascular diseases (4-6). Diagnosis of the disease in early stages followed by precise control of blood glucose (7-9) in combination with blood pressure controlling by suitable medications (angiotensin converting enzyme inhibitors and angiotensin receptor blockers) may prevent progress of microalbuminuria to macroalbuminuria and that may cause further prevention from renal failure (10-13).

In order to detect nephropathy, measurement of total (24 hrs) urinary albumin or albumin/creatinin ratio in random urine samples is being recommended (1). There are few studies that have examined relationship between total urinary protein and albumin and diagnosis of albuminuria, however some studies (14-18) have suggested measurement of total urinary protein as an appropriate alternative to detect urinary albumin for macroalbuminuria diagnosis. As methods of albumin measurement are not available in all laboratories and also cost about 6 times more than that of urinary total protein measurement, the latter method is an easier, more available, and less expensive method.

## 2. Objectives

The purpose of this study was determination an appropriate 24 hrs urine protein cut off point to diagnose microalbuminuria and macroalbuminuria by keeping urine albumin as the gold standard.

## 3. Patients and Methods

In this study, all patients with diabetes mellitus (type I and type II) who were referred to endocrine clinic of Boo-Ali Hospital of Qazvin city from September 2008 to April 2009 were studied. A questionnaire, including age, sex, medical condition, and medication intake especiallyangiotensin converting enzyme (ACE) inhibitors and Aldosteron receptor blockers (ARB) was completed. Patients with renal disease caused by nondiabetic etiologies such as congestive heart failure, urinary tract infection, or hematuria were excluded. 24 hour urine samples were collected to analyze total protein and total albumin. Urine albumin was measured by a non-linear immunoturbidimetry method used in automated Pars Azmoon kit with accuracy of 3 mg/day. Urinary albumin of 30 mg/day was considered as normal urinary albumin exertion. Urine protein was analyzed by Pyrogallol colorimetric method by manual using of Shym enzyme kit and spectrophotometer. Data were tested for normal distribution prior to performing statistic parameters and reported as mean ± SDs. Correlation between urinary protein and urinary albumin was measured by correlation Pearson test. The comparison between albumin/protein ratio among 3 groups of normo-, micro- and macroalbuminuric patients was performed by ANOVA statistical test. Using receiver operating curve (ROC), areas under the curves were calculated to find out appropriate urine total protein cut off point for diagnosis micro- and macroalbuminuria. SPSS version 15 was used for analysis.

## 4. Results

All 204 patients having specifications shown in Table 1 completed the study. Normoalbuminuric ( < 30 mg/day), microalbuminuric (30-300 mg/day), and macroalbuminuric ( > 300 mg/day) measures were found in 130, 51, and 23 patients, respectively (Table 1).

**Table 1:** Specifications of Patients

	Normoalbuminuria < 30 mg/Day	Microalbuminuria 30-300 mg/Day	Macroalbuminuria > 300 mg/Day	Total
Number of patients	130	51	23	204
Diabetes Mellitus Type I	23	4	3	30
Diabetes Mellitus Type II	107	47	20	174
Duration of disease, y	7.6 ± 0.2 a	8.5 ± 6.3 a	13.3 ± 10.1 a	8.4 ± 7 a

^a^Mean ± SD

Patients’ mean urine albumin vs. protein in normo-, micro-, and macroalbuminuria groups were 8.64 ± 0.77 vs. 58.75 ± 2.36, 122.18 ± 30.36 vs. 275.02 ± 64.28, and 394.29 ± 80.8, 594.26 ± 303.2 respectively. (Table 2).

**Table 2:** Urine Albumin and Protein Correlation Among Groups of Study

	Normoalbuminuria	Microalbuminuria	Macroalbuminuria
Albumin mg/day	8.64 ± 0.77 a	122.18 ± 30.36 a	394.29 ± 80.8 a
Protein mg/day	58.75 ± 2.36 a	275.02 ± 64.28 a	594.26 ± 303.2 a
Albumin/protein	0.16 ± 0.01 a	0.44 ± 0.2 a	0.46 ± 0.03 a
Correlation (r)	0.26 (*P* < 0.01)	0.97 (*P* < 0.001)	0.91 (*P *< 0.001)

^a^Mean ± SD

Urinary albumin/protein ratios shown in Table 2 among micro- and macroalbuminuria groups were higher than that in normoalbuminuric patients in different stages of nephropathy (0.44 0.02 ± and 0.46 0.03 ±, respectively versus 0.16 0.01 ±, P < 0.0001). Correlation of total urinary protein with urinary albumin was significant in each of three groups of normo-, micro-, and macroalbuminuria (r = 0.26, P < 0.01; r = 0.97, P < 0.001; and r = 0.91, P < 0.001, respectively).

**Figure 1:**
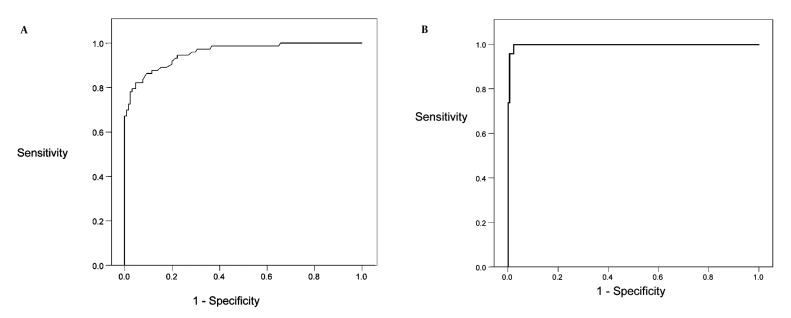
Urinary Total Protein in Microalbuminurea ROC (Receiver Operating Characteristics) curves displaying the relationship between sensitivity and specificity that defined urinary total protein in microalbuminuric (A, area under the curve 0.957 ± .014) and macroalbuminuric patients (B, area under the curve, 0.998 ± .002)

Using ROC curves and calculating under the curve area, urinary total protein cut off point of 73 mg/day had an appropriate sensitivity and specificity to detect microalbuminuria (area under the curve 0.957 0.014 ±; sensitivity 94.5 %, CI = 91.4 %-97.6 %; and specificity 77.9 %, CI = 72.8 %-82.9 %) (Figure 1A). Also, value of 514 mg/day was detected as an appropriate cut off point for diagnosis macroalbuminuria (area under the curve 0.998 0.002 ±; sensitivity 95.7 %, CI = 92.9-98.5 % ;and specificity 98.9 %, CI = 96.8-100 %) (Figure 1B). The urine protein of 150 mg/day which is currently considered as a normal value for urine protein exertion in most laboratory kits had a sensitivity of 73.1 %, CI = 63.9-82.3 % that means about 30 % of microalbuminuric patients remain undiagnosed. Comparing patients taking ACE or ARB drugs, there were no significant differences in sensitivity and specificity of different urinary total protein cut off points. Only in urine total protein cut off point of 73 mg/day, there was a significant difference in specificity among patients managed with or without the drugs; in drug managed patients, specificity reduced to 68.4 %versus 81.7 % in non-drug managed patients, (P < 0.05) (Table 3). In other word, about 32 % of normal subjects were not detected.

**Table 3:** Comparison of Sensitivity and Specificity of Different Urine Total Protein Cut off Points for Diagnosis Micro- and Macroalbumiuria Among Medication (ACE & ARB) Users and Non-Users

Cut Off Points mg/day	ACE & ARB Users (n = 52)	ACE & ARB Non-Users	Total Patients	*P* value
**Sensitivity**				
44.5	100 %	100 %	100 %	NS
73	96.2 % (92.2-100) a	90.5 % (85.1-95.9)	94.5 % (91.4-97.6)	NS
107	88.5 % (81.9-95.1)	66.7 % (58.1-75.3)	82.2 % (77.5-87.9)	NS
150	73.1 % (63.9-82.3)	61.9 % (52.9-70.8)	69.9 % (63.6-76.2)	NS
418			100 %	
514			95.7 % (92.9-98.5)	
694			73.7 % (67.7-79.3)	
**Sensitivity**				
44.5	23.7 % (14.9-32.5)	38.7 % (29.8-47.6)	34.0 % (27.5-40.5)	NS
73	68.4 % (58.8-78.0)	81.7 % (74.6-88.8)	77.9 % (72.8-82.9)	*P* < 0.05
107	86.8 % (79.8- 93.8)	97.8 % (95.1-100)	94.75 (91.6-96.7)	NS
150	97.4 % (94.1-100)	100 %	99.2 % (97.8-100)	NS
418			97.8 % (95.8-100)	
518			98.9 % (96.8-100)	
694			100 %	

^a^CI %, ACE & ARB users (n = 52) and non-users (n = 21) in microalbuminuria groups. ACE & ARB users (n = 18) and non-users (n = 5) in macroalbuminuria groups. Due to small size of ACE & ARB non-users (n = 5) in macroalbuminuria group, comparison was not performed.

## 5. Discussion

Our study revealed that urinary total protein cut off point of 73 mg/day had appropriate sensitivity and specificity to detect microalbuminuria. Also, the amount of 514 mg/day total protein was detected as a proper cut off point to diagnose macroalbuminuria. Comparing medicated and non-medicated patients with ACE and/or ARB drugs having similar urinary total protein of 73 mg/day, there was a significant difference in specificity of the test between these two groups.

The detected urinary total protein cut off point demonstrated in our study to use formicroalbuminuria diagnosis was significantly different with the value routinely considered asnormal urinary total protein in most laboratory kits (73 mg/day versus 150 mg/day). Urinary protein of 150 mg/day had sensitivity of 73.1 % ; this results in undiagnosis of about 30 % of microalbuminuric patients. However, our finding about urinary total protein cut off point for macroalbuminuria dtection was almost similar to that of previous norm (514 mg/day versus 500 mg/day).

Zelmanovitz et al. (18) have suggested the amount of 541 mg/day of urine total protein (sensitivity 100 %, specificity 95.7 %) as an appropriate cut off point for diagnosis macroalbuminuria which is comparable with our finding of 514 mg/day (sensitivity 95.7 %, specificity 98.9 %) having significant accuracy of 98.5 % .

In Khatami et al study (15), urine protein cut off value of 24 mg/lis reported as the best value for detecting microalbuminuria (sensitivity and specificity 86 % and 90 %, respectively). While in this study urine protein value was reported in the form of concentration (mg/l), in gold standard (urine microalbumin) it is determined by albumin/creatinin ratio. So, the difference between best cut off point in our study and Khatami et al. study could be related to this point.

In our current study, there was a strong relationship between urinary albumin and protein levels in micro- and macroalbuminuria groups. The ratio of urinary albumin to total protein in micro- and macroalbuminuria groups were 0.44 ± 0.02, r = 0.97 and 0.46 ± 0.03, r = 0.91, respectively (P < 0.001). Our study confirmed previous studies (14, 16, 17) that reported strong relationship between urinary albumin and protein.

Considering various ethiologies of proteinuria in nephropathies, results of this study would be generalized only to diabetic patients with micro- and macroalbuminuria.

In conclusion, 24 hours urine total protein might be used as a diagnostic indicator applied with much lower costs for detection micro- and macroalbuminuria in diabetic patients.
